# Protein kinase C inhibitors override ZEB1-induced chemoresistance in HCC

**DOI:** 10.1038/s41419-019-1885-6

**Published:** 2019-09-23

**Authors:** Rahul Sreekumar, Muhammad Emaduddin, Hajir Al-Saihati, Karwan Moutasim, James Chan, Marcello Spampinato, Rahul Bhome, Ho Ming Yuen, Claudia Mescoli, Alessandro Vitale, Umberto Cillo, Massimo Rugge, John Primrose, Mohammad Abu Hilal, Stephen Thirdborough, Eugene Tulchinsky, Gareth Thomas, Alex Mirnezami, A. Emre Sayan

**Affiliations:** 1 University of Southampton Cancer Sciences Division, Somers Cancer Research Building, Southampton University, Tremona Road, Southampton, UK; 2 Department of Surgery, Southampton University Hospital NHS Trust, Southampton, UK; 3 HPB Unit, Department of General and Minimally Invasive Surgery, Policlinico of Abano Terme, Abano Terme, Italy; 4 Primary Care and Population Sciences, University of Southampton, Southampton, UK; 5 Department of Pathology, University of Padua, Padua, Italy; 6 Hepatobiliary and Liver Transplantation Unit, University of Padua, Padua, Italy; 7 Cancer Sciences and Molecular Medicine Department, University of Leicester, Leicester, UK; 8 Moscow Institute of Physics and Technology, Dolgoprudnuy, Moscow region, Moscow, Russia; 9 Department of Biomedical Sciences, Nazarbayev University School of Medicine, Astana, Kazakhstan

**Keywords:** Metastasis, Predictive markers

## Abstract

Epithelial–mesenchymal transition (EMT) is a process by which tumour cells lose epithelial characteristics, become mesenchymal and highly motile. EMT pathways also induce stem cell features and resistance to apoptosis. Identifying and targeting this pool of tumour cells is a major challenge. Protein kinase C (PKC) inhibition has been shown to eliminate breast cancer stem cells but has never been assessed in hepatocellular cancer (HCC). We investigated ZEB family of EMT inducer expression as a biomarker for metastatic HCC and evaluated the efficacy of PKC inhibitors for HCC treatment. We showed that ZEB1 positivity predicted patient survival in multiple cohorts and also validated as an independent biomarker of HCC metastasis. ZEB1-expressing HCC cell lines became resistant to conventional chemotherapeutic agents and were enriched in CD44^high^/CD24^low^ cell population. ZEB1- or TGFβ-induced EMT increased PKCα abundance. Probing public databases ascertained a positive association of ZEB1 and PKCα expression in human HCC tumours. Inhibition of PKCα activity by small molecule inhibitors or by *PKCA* knockdown reduced viability of mesenchymal HCC cells in vitro and in vivo. Our results suggest that ZEB1 expression predicts survival and metastatic potential of HCC. Chemoresistant/mesenchymal HCC cells become addicted to PKC pathway and display sensitivity to PKC inhibitors such as UCN-01. Stratifying patients according to ZEB1 and combining UCN-01 with conventional chemotherapy may be an advantageous chemotherapeutic strategy.

## Introduction

Hepatocellular carcinoma (HCC) is a common and deadly cancer^1^. HCC is very resistant to cytotoxic chemotherapy, therefore most patients are treated with surgery or ablation^2^. However, such approaches usually fail due to the presence of advanced disease at presentation^3^. Most patients who undergo curative surgery subsequently develop intra- and extra-hepatic metastases. Currently, there are no biomarkers that can prognosticate HCC trajectory or drugs that can target chemoresistant/metastatic cells.

EMT is a *trans*-differentiation programme that plays a major role in cancer spread by inducing the formation of motile/metastatic carcinoma cells^4^. EMT pathways also facilitate acquisition of stem cell properties and chemoresistance^4–6^. Diverse extracellular stimuli activate EMT pathways by inducing Twist, SNAIL and the ZEB family of transcription factors (EMT-TFs)^6^. As EMT-TF’s are initiators of EMT programmes, identifying the correct EMT inducer and using its expression as a biomarker will allow patient stratification for defining metastatic potential and also decision for therapy. Among EMT-TFs, ZEB family members have not been studied in a collective manner in HCC. As metastasis and therapy-resistance represent the principal causes of HCC-related mortality, understanding the expression and function of ZEB proteins is critically important.

Here we evaluate the expression of E-Cadherin, ZEB1 and ZEB2 in primary HCC. We showed that ZEB1, but not ZEB2, positivity predicted poor patient survival and externally validated it as a biomarker of HCC metastasis using two independent patient cohorts. We also assessed the functional contribution of ZEB1 to EMT, chemoresistance and hepatosphere forming properties. Recently, protein kinase C alpha (PKCα) activation has been implicated in the formation and survival of cancer stem cells (CSCs)^7^. It also became evident that mesenchymal/metastatic carcinoma cells are not addicted to Ras oncogene^8^ but require PKC pathway activation for survival^7,8^. Therefore, exploring in vitro and in vivo models, we asked whether PKC pathway is implicated in the survival of ZEB expressing HCC cells.

## Materials and methods

Please see Supplementary information for assessment of cell viability, apoptosis and motility, expression analysis, western blotting and immunofluorescence, hepatosphere formation assay and bioinformatic analysis.

### Patient material and analysis of ZEB immunoexpression

Paraffin-embedded samples of primary HCC were included from two prospectively maintained registries of consecutive patients, who underwent tumour resection between 1997 and 2010 at the Department of Surgery, University of Southampton, UK, and 96 consecutive patients who underwent tumour resection between 2001 and 2006 from the Department of Surgery, Policlinico di Abano Terme, Padua, Italy. Patient anonymization and IHC was performed using ethics no: 10/H0504/32. Results are transparently presented according to the Biospecimen Reporting for Improved Study Quality (BRISQ) and reporting recommendations for tumour marker prognostic studies (REMARK) guidelines^9,10^. Median follow-up time was 21 months for the UK cohort and 60 months in the Italian cohort. Tumour containing blocks were cut to (4 μM) sections and immunohistochemical staining for ZEB1, ZEB2 and E-Cadherin undertaken as previously described^11^. Further details of this section can be found in Supplementary information.

### Statistical analysis

SPSS version 21.0 (SPSS, Chicago, IL, USA) was used for all statistical analysis. Two sample *t*-test was performed for analysis of migrations and viability assays. Chi-square or Fisher’s exact test (where appropriate) was applied to assess independence of ZEB1, ZEB2 and E-Cadherin expression with clinic-pathological parameters. Kaplan–Meier survival curves were used to assess differences in OS and DFS, and significance reported using the Log-rank test. Multivariable analysis was performed using Cox-proportional hazards regression and included all the covariates listed in Supplementary Table S1. The survival end-point for univariate and multivariable analysis was time to recurrence (DFS) or time to death (OS) after surgery. Patients with missing outcome data were right-censored from analysis with the last observed outcome. Student *t*-test was performed during gene expression analysis and group comparisons, considering the groups are not paired. In all statistical analysis results were considered significant when the *p*-value was <0.05.

### Cell lines, transfections and reagents

SNU387, SNU423, SNU475, Huh7, PLC/PRF/5 (HCC), HepG2 (Hepatoblastoma) and SKHep1 (adenocarcinoma of liver) were purchased from ATCC. Hep40 cells (HCC) were kindly provided by Prof. M. Ozturk (IBG, Izmir, Turkey). Cells were propagated in DMEM (PAA) supplemented with 10% FCS, Penicillin/Streptomycin (50 U/ml) and 2 mM L-Glutamine in a humidified, 5% CO_2_ incubator. The authenticity of Hepatoma cell lines were validated by STR analysis (Eurofins, Germany) and sequencing of p53 cDNA as they contain different p53 mutations^12^. MycoAlert Mycoplasma Detection Kit (Lonza) has been used routinely to check mycoplasma contamination. Full-length human ZEB1 and ZEB2 cDNA were cloned into pCDNA4 plasmid with N-terminal HA-tag and sequence verified. E-Cadherin promoter luciferase reporter was used as described before^11^. Transfections were performed using Lipofectamine LTX reagent (Invitrogen). Where necessary, cells were treated with TGFβ (R&D systems), UCN-01 and Midostaurin (Enzo Lifesciences), Oxaliplatin (Hospira, UK) and Sorafenib (Bayer, UK). All other chemicals were obtained from Sigma. Small hairpin RNA constructs (control and validated PKCα targeting, TRC no: TRCN0000195322-PKC-sh-1 and TRCN0000001693-PKC-sh-2) were purchased from Sigma.

### In vivo studies

Female SCID BALB/C mice (12–14-week old) were used in all in vivo experiments. For subcutaneous injections, 1 × 10^5^ cells (SKHep1, Huh7, PLC/PRF/5 or HepG2) were mixed 50/50 with Matrigel (BD) and injected as 100 μl on both flanks. Ten days later, and when tumours became palpable, UCN-01 (2 mg/kg) or PBS was injected intra-peritoneally weekly for the duration of the experiment. The follow-up time was set to 14 weeks for the experiment involving SKHep1 and 6 weeks for E-HCC cells. All animals were independently assessed by animal facility technicians for welfare reasons and culled when tumour burden exceeded pre-defined acceptable limits. For orthotopic injections 2.5 × 10^5^ SNU387 or SKHep1 cells were mixed 50/50 with Matrigel and injected as 60 μl into liver parenchyma after laparotomy. Four animals were assigned to each group (control vs UCN-01). Animals were allowed to recover from surgery for 10 days before intraperitoneal UCN-01 (2 mg/kg/week) or PBS (control) treatments. Animals were assessed and weighed weekly by researchers blinded to the treatment groups. When weight loss exceeded 20% (week 5 for SKHep1, week 7 for SNU387), all animals were injected intravenously with the 800CW-2DG probe (Licor), and culled 18 h later. Livers and lungs were harvested, imaged and analysed using an IVIS Lumina III imaging unit (Perkin Elmer). The unit of signal is set to average radiant efficiency (Fluorescence emission radiance per incident excitation power) as recommended by the manufacturer.

## Results

### Assessment of ZEB family protein expression in HCC

**Fig. 1:**
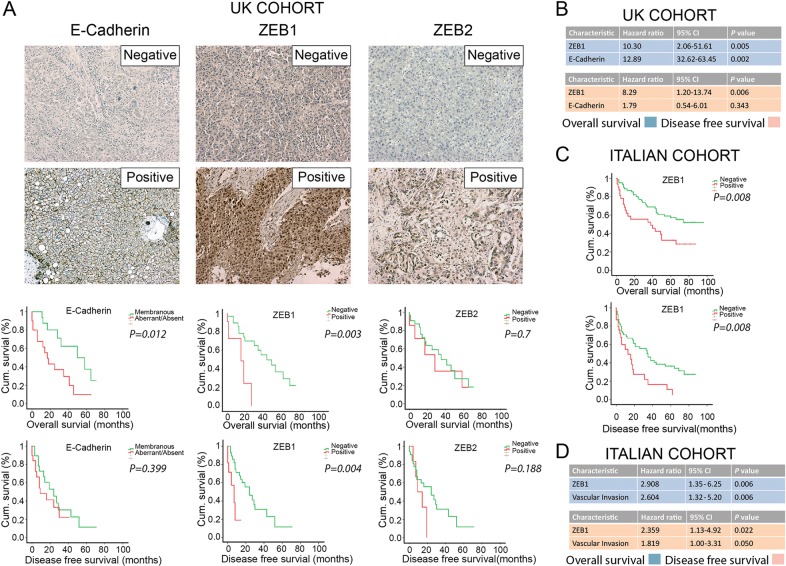
Immunoexpression of ZEB family proteins in primary HCC is associated with outcome in independent patient cohorts from the UK and Italy. **a** E-Cadherin (left panels), ZEB1 (middle panels) and ZEB2 (right panels) expression in UK-HCC cohort (*n* = 40) as detected by IHC. Considering the functions of the proteins, membranous E-Cadherin expression is marked as positive whereas cytoplasmic (aberrant) and no-expression of E-Cadherin is marked as negative. More than 10% nuclear ZEB1 or ZEB2 expression is marked as positive, <10% or predominant cytoplasmic expression is marked as negative. Absence of E-Cadherin or presence of ZEB1 is associated with overall survival whereas ZEB1 positivity is associated with disease-free survival (time to metastasis/recurrence). **b**, **d** Independent prognostic markers, as assessed by Cox regression analysis, showing statistical significance in the UK (**b**) and Italian (**d**) cohorts. Blue charts indicate overall survival; pink charts indicate disease-free survival. CI: confidence interval. **c** ZEB1 expression is significantly associated with both overall and disease-free survival in Italian-HCC cohort (*n* = 96)

The functional redundancy of ZEB family in relation to patient survival was not studied in HCC, therefore we investigated the expression of E-Cadherin, ZEB1 and ZEB2 by IHC, using 40 consecutive HCC patients operated in the UK. Clinico-pathological variables of this cohort are provided in Table S1. Predominant nuclear expression of ZEB1 is observed in 28% of tumours (Fig. 1a). ZEB1 expression was significantly associated with vascular invasion, tumour stage, and presence of satellite lesions (Supplementary Table S1). Median overall survival (OS, *p* *=* 0.003) and disease-free survival (DFS, *p* *=* 0.004) were reduced in the ZEB1-positive group (Fig. 1a). Multivariate analysis revealed ZEB1 expression as a significant independent marker of poor OS (*p* *=* 0.005) and DFS (*p* *=* 0.006) (Fig. 1b). ZEB2 positivity was detected in 18% of samples (Fig. 1a). No correlation was observed between ZEB2 expression and OS or DFS. E-Cadherin expression was associated with OS (*p* *=* 0.012) but not with DFS (Fig. 1a). Multivariate analysis demonstrated aberrant/negative E-Cadherin expression as an independent prognostic factor of OS (*p* *=* 0.002), but not DFS (Fig. 1b). Other variables did not associate with any proteins investigated (Supplementary Table S1).

EMT-TF expression was linked with clinical outcomes previously, however, to date, no EMT-associated biomarker has entered clinical practice due to lack of external validation. To address this, we further analysed 96 consecutive HCC samples from an independent patient cohort (Italy). Clinico-pathological characteristics for this cohort are presented in Supplementary Table S2. ZEB1 expression was associated with decreased OS (*p* *=* 0.008) and DFS (*p* = 0.008) but not with other variables (Fig. 1c and Supplementary Table S2). Presence of ZEB1 or vascular invasion was independent prognostic markers of OS and DFS (Fig. 1d). Transparent reporting of biospecimen details, patient cohorts, variables evaluated, and statistical analyses for both cohorts are detailed in Supplementary Tables S1–3 in compliance with BRISQ and REMARK guidelines^9,10^. The observation that ZEB1 expression predicts HCC recurrence and patient survival in multiple independent cohorts strengthens the argument for its use as a prognostic biomarker in HCC.

### Functional role of ZEB proteins in HCC

**Fig. 2:**
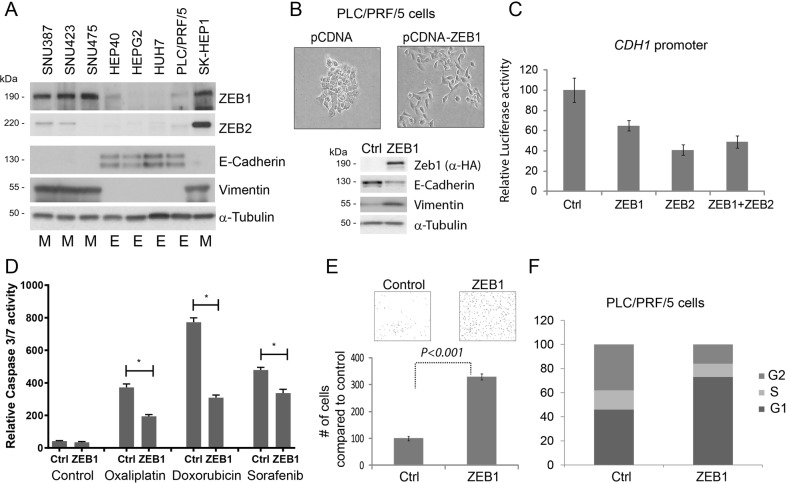
Transcription factors of ZEB family are expressed in HCC-derived cell lines and contribute to epithelial plasticity. **a** Expression of ZEB1, ZEB2, E-Cadherin and vimentin proteins was assessed by western blotting in eight Hepatoma-derived cell lines. Cell lines identified as epithelial are marked with “E”, mesenchymal with “M”. **b** Transient expression of ZEB1 induced cell scattering and affected canonical markers of EMT in PLC/PRF/5 cells such as increased vimentin and decreased E-Cadherin protein expression. **c** Both ZEB1 and ZEB2 suppressed *CDH1* promoter in transient reporter assay. **d** ZEB1-induced EMT facilitates resistance to apoptosis to commonly used chemotherapeutic agents used in HCC treatment. Cells were treated with 100 μM Oxaliplatin (Ox), 2 μg/ml Doxorubicin (Dox) and 10 μM Sorafenib (Sor) for 24 h. Arbitrary units of luciferase activity defining apoptosis (caspase 3/7 activity) has been presented. In all cases, ZEB1-expressing cells became resistant to cell death. (*) is *p* < 0.05 as assessed by Student’s *t*-test. **e** ZEB1-induced EMT facilitates cell motility as assessed by transwell-migration assay. PLC/PRF/5 cells expressing ZEB1 are significantly (approximately three times) more motile compared with control. **f** Assessment of cell cycle profile 3 days after ZEB1 overexpression revealed an enrichment of G1 cells indicating cell cycle arrest

Our findings suggest acquired ZEB1 expression predicts clinical outcome in HCC, therefore we investigated its functional contribution towards HCC biology. Initially we analysed ZEB1, ZEB2, E-cadherin and vimentin expression in eight Hepatoma-derived cell lines. Four of eight cell lines were morphologically epithelial and expressed E-Cadherin (E-HCC); the remainder grew as single cells, expressed vimentin and were mesenchymal (M-HCC, Fig. 2a). We observed an inverse-correlation between the expression of E-Cadherin and ZEB1/ZEB2, further supporting a role for ZEB family as critical EMT-inducers in HCC. Overexpression of ZEB1 activated EMT, as PLC/PRF/5 cells became mesenchymal, downregulated E-Cadherin and increased vimentin (Fig. 2b). Both ZEB family members suppressed *CDH1* promoter-driven luciferase expression (Fig. 2c). Ectopic ZEB1 expression induced chemoresistance to chemotherapeutics used in HCC treatment (Fig. 2d) and significantly increased motility of PLC/PRF/5 cells (Fig. 2e) in agreement with our in vivo observations that ZEB1 immunoexpression is associated with metastatic phenotype. ZEB1 also induced a partial G1-arrest, which is considered a hallmark of EMT (Fig. 2f)^13^.

### Mesenchymal features define chemoresistance and stem cell ability of HCC

**Fig. 3:**
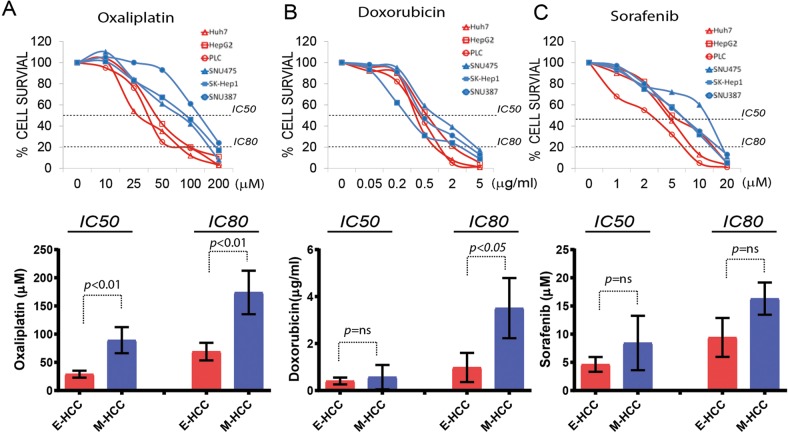
Chemoresistance profiles of Hepatoma cells associate with mesenchymal properties. The set of three epithelial (Huh7, PLC/PRF/5, HepG2) and three mesenchymal (SKHep1, SNU387, SNU475) Hepatoma-derived cell lines were treated with Oxaliplatin, Doxorubicin or Sorafenib for 8 h, and viability was assessed by 96 h after recovery. Mean IC value for each set was presented in the graph below. *P* values more than 0.05 are considered not significant using *IC50* and *IC80* values presented in Supplementary Fig. 1A and calculated by unpaired Student *t-*test. **a** E- and M-HCC cells showed stratification upon Oxaliplatin treatment at the *IC50* and *IC80* values. **b** Doxorubicin *IC80*, but not *IC50*, value marks significance difference between two groups. **c** EMT status did not correlate with Sorafenib toxicity at *IC50* or *IC80*

A feature of EMT is promoting resistance to apoptosis, therefore chemotherapy^5^. We already ascertained that ZEB1 overexpression induces resistance to apoptosis in HCC (Fig. 2d); however, mutation burden may be defining sensitivity to chemotherapeutic agents. We, therefore, treated six Hepatoma cell lines that are genetically different, and representing epithelial (*n* = 3) or mesenchymal (*n* = 3) morphology, with three commonly used chemotherapeutic agents. M-HCC cells were chemoresistant to Oxaliplatin both at *IC50* and *IC80* values (Fig. 3a, Supplementary Fig. S1). Considerable percentage of M-HCC cells survived higher doses Doxorubicin (Fig. 3b) creating a significant difference in *IC80* values (Fig. 3b, Supplementary Fig. S1). Apart from Oxaliplatin and Doxorubicin, the Sorafenib is increasingly used in HCC treatment^14^. Cell lines displayed no trend in terms of Sorafenib-related toxicity and EMT status (Fig. 3c, Supplementary Fig. S1). These findings suggest that genetically identical (control vs ZEB1 overexpressing cells, Fig. 2d) or genetically different but morphologically similar Hepatoma cells (Fig. 3a–c) can be stratified according to their EMT status and chemoresistance. Therefore, treatment of metastatic HCC with DNA damaging agents is not an effective therapeutic strategy.

**Fig. 4:**
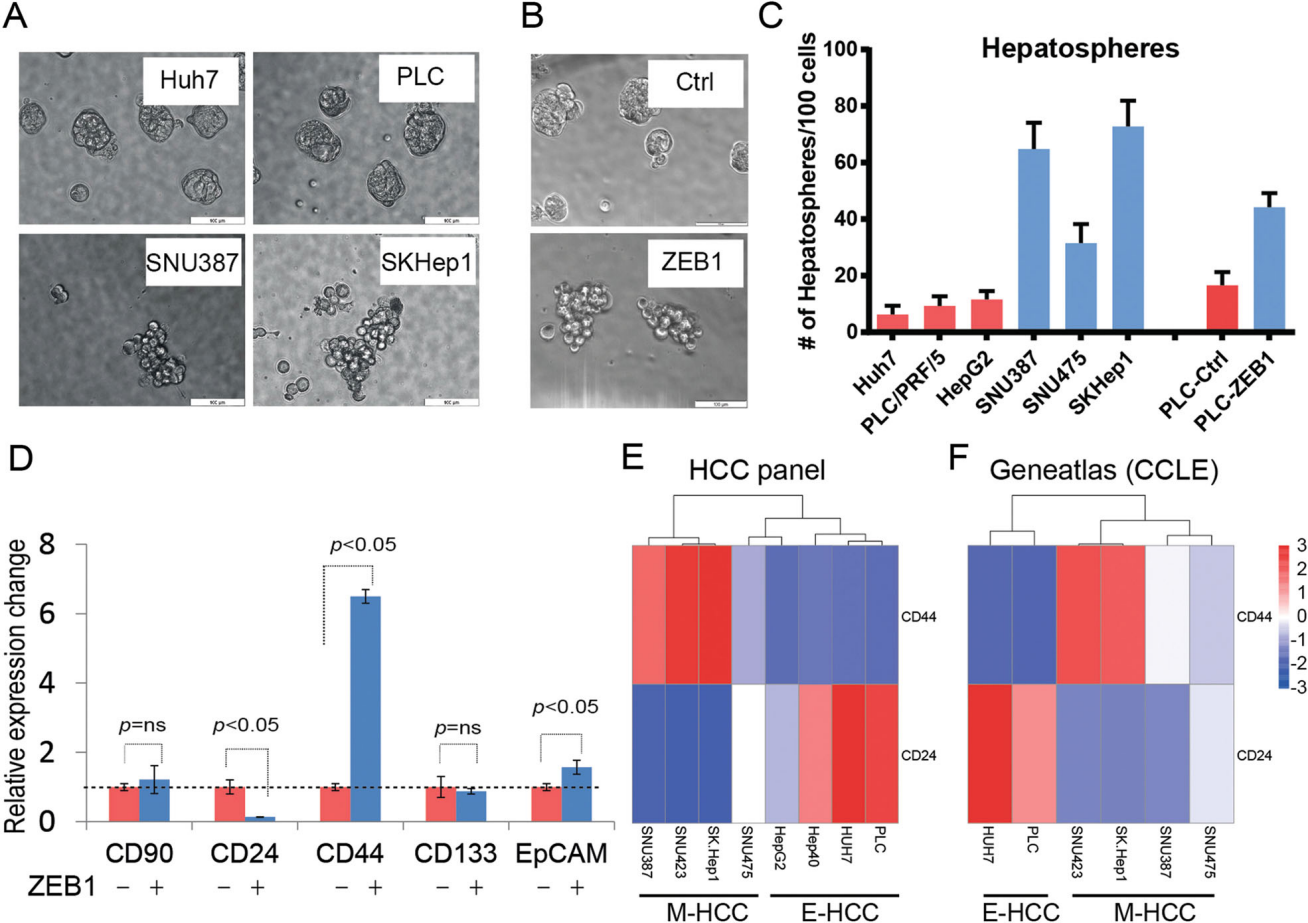
M-HCC phenotype is associated with enhanced hepatosphere formation and CD44^high^/CD24^low^ profile. **a** In conditions of non-adherent growth, E-HCC cells produced spherical and smooth hepatospheres (top panels). M-HCC cells (bottom panels) produced loosely attached cell clusters in the shape of a grape bunch. **b** ZEB1-induced EMT mimics the phenotype observed in M-HCC cells. **c** E-HCC and M-HCC cells show stark differences in terms of number of hepatospheres formed. M-HCC cells or EMT phenotype induced by ZEB1 increases the number of hepatospheres formed indicating they contain more stem cell-like cells. **d** A panel of currently used stem cell markers were assessed by qPCR in HCC upon ZEB1 overexpression. The changes in CD44, EpCAM and CD24 were significant. Among the changing ones CD44 (increase) CD24 (decrease) were most prominent. The analysis of CD44 and CD24 in E- and M-HCC cells by qPCR (**e**) or RNA-seq by probing geneatlas database (**f**). The expression data in the form of ΔΔcT (**e**) or “reads” (**f**) were presented after non-hierarchical cluster analysis. E- and M-HCC cells clustered as a result of CD24 and CD44 expression

EMT pathways are known to induce enrichment of CSC subpopulations^15^. We, therefore, examined sphere-forming ability of Hepatoma cell lines and upon ZEB1 overexpression. E-HCC cell lines formed tightly clustered spheres whereas M-HCC cells, or ZEB1 overexpressing PLC/PRF/5 cells formed clusters similar to grape bunches (Fig. 4a, b). Importantly, the number of hepatospheres was significantly more in M-HCC cells or upon ZEB1 expression compared with epithelial counterparts (Fig. 4c). Further, we investigated the expression of previously proposed stem cell markers such as CD133, CD90, CD24, CD44 or EpCAM^16^ during ZEB1-induced EMT. ZEB1 overexpression facilitated a significant change in CD24 and CD44 abundance, whereas all the other potential markers were unchanged (CD90 and CD133) or changed significantly (EpCAM) but remained barely detectable (Fig. 4d). In breast cancer (BC), CD44 +/CD24^low^ phenotype dictates stem cell status, induced by EMT-TFs, and is enriched in mesenchymal/chemoresistant BC cell lines^7,17^. Expression analysis followed by non-hierarchical clustering analysis of E- and M-HCC cell lines for CD24 and CD44 expression confirmed that reduction in CD24 and increase in CD44 (Fig. 4e). The abundance of CD24 and CD44 in Hepatoma cell lines have also been reported in CCLE and Genentech cohorts of geneatlas database and concur with our observations (Fig. 4f and Supplementary Fig. S2)^18^. Supporting our data, an association of mesenchymal gene expression signature (Twist+, Albumin−, AFP−) with CD44 increase was previously reported in HCC^19^. These results suggest CD24^low^/CD44^high^ Hepatoma cells display mesenchymal phenotype, express ZEB1, are stem cell-like, motile and chemoresistant.

### Protein kinase C (PKC) inhibitors selectively kill M-HCC cells

**Fig. 5:**
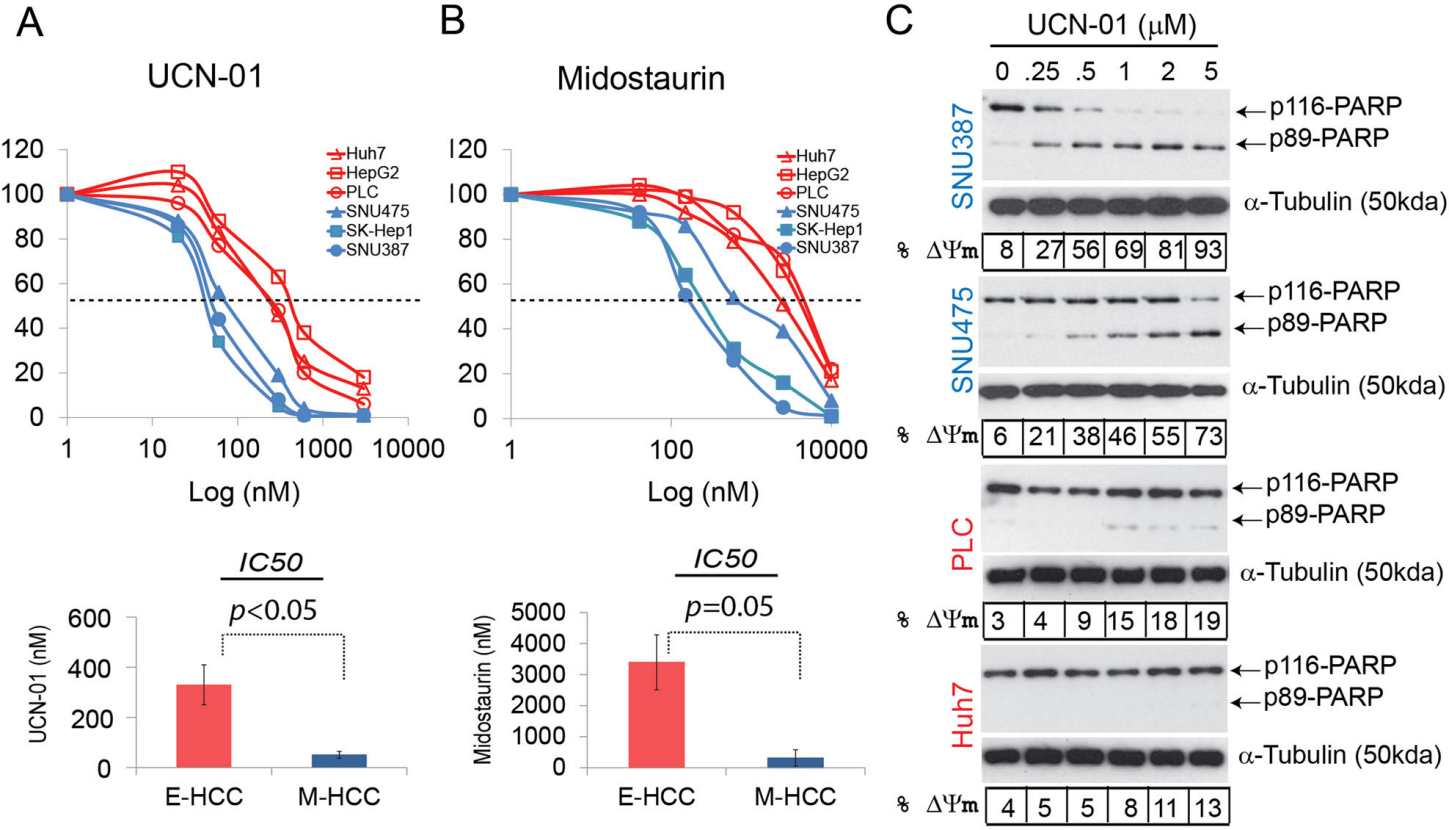
Hepatoma cells respond to PKC inhibitors according to their EMT status. Viability assays defining *IC50* concentrations of UCN-01 (**a**) and Midostaurin (**b**) show that E- and M-HCC cells are stratified in their responses. The mean *IC50* of E-and M-HCC cells were significantly different for all PKC inhibitors. Student’s *t*-test was used to identify the significance of tested groups. **c** The activity of UCN-01 was assessed in mesenchymal (SNU387 and SNU475) and epithelial (PLC/PRF/5 and Huh7) cells. All cell lines were treated with increasing concentrations of UCN-01 (0.25–5μM) for 8 h. The cells were collected and sample divided into 2 for western blotting (PARP cleavage) and mitochondria depolarization (% ΔΨm) to confirm hallmarks of apoptosis. UCN-01 induced substantial apoptosis only in mesenchymal HCC cells

Cancer-EMT creates a spectrum of phenotypes^20^ therefore it is common to observe epithelial, mesenchymal or partial-EMT features within one tumour^21^. We have shown current chemotherapy regimens for HCC impact E- but not M-HCC cells. For greater clinical efficacy, however, both epithelial- and mesenchymal-cell components of HCC need to be targeted. A recent article identified PKC inhibitors as having selective efficacy in killing BC stem cells^7^. Accordingly, we tested the response of the E- and M-HCC cells to clinically trialed PKC inhibitors, Midostaurin and UCN-01^22,23^. *IC50* curves revealed that significantly lower concentrations of UCN-01 and Midostaurin were inhibiting the viability of M-HCC, as compared with E-HCC cells (Fig. 5a, b). The mean *IC50* for E- and M-HCC cells were significantly different for both drugs (Fig. 5a, b lower panels and Supplementary Fig. S3A). M-HCC cells showed extensive apoptosis as assessed by PARP cleavage and mitochondrial depolarization upon an 8 h UCN-01 treatment whereas limited/no apoptosis was observed in E-HCC cells (Fig. 5c). On the other hand, a longer treatment (36 h) and higher concentrations of Midostaurin were required to observe detectable apoptosis in M-HCC cells (Supplementary Fig. S3B). Unlike UCN-01, Midostaurin induced polyploidy in all cells tested including non-transformed cells such as fibroblasts (Supplementary Fig. S4A). All PKC inhibitors induced apoptosis in M-HCC cells and exhibited limited/no activity in E-HCC cells at tested conditions, suggesting their action represents class effect (Fig. 5 and Supplementary Figs. S3–4). Importantly, normal mesenchymal cells such as fibroblasts tolerated UCN-01 better than M-HCC cells (Supplementary Fig. S4B). Taken together, our data suggest UCN-01 and Midostaurin are effectively killing chemoresistant/mesenchymal HCC cells.

**Fig. 6:**
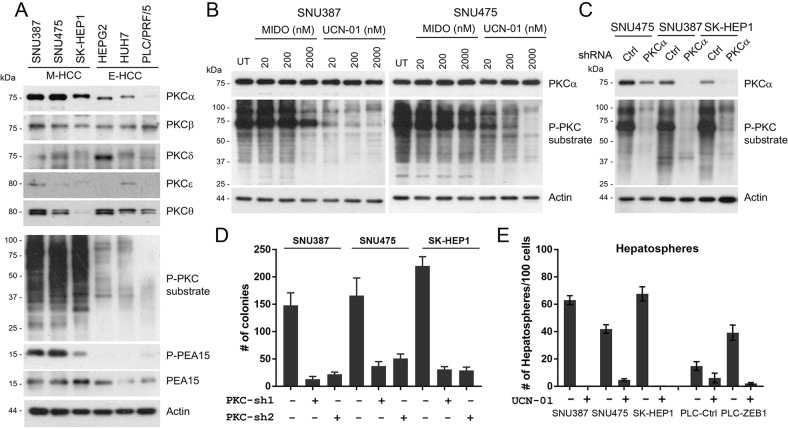
PKC pathway is activated in M-HCC cells and necessary for M-HCC survival. **a** The expression of different PKC family proteins and PKC activity was investigated in a panel of HCC cell lines. PKC pathway activation in M-HCC cell lines was evident as pan phospho-PKC-substrate antibody gave strong reactivity along with a specific PKCα substrate such as PEA-15. Among different PKCs, only the expression of PKCα correlated with mesenchymal status (M-HCC cells). Other PKC family proteins were either uniformly expressed or not correlated with EMT status of HCC cell lines. **b** A dose-escalation study using two M-HCC cell lines revealed UCN-01 as more effective in inhibiting PKC activity as compared with Midostaurin. In both cell lines 20 nM UCN-01 inhibited PKC phosphorylation better than 2 μM of Midostaurin. **c** Transient knockdown of PKCα effectively inhibited the PKC activity in three M-HCC cells confirming that PKCα is responsible for the observed PKC activity in M-HCC cells. **d** Stable knockdown of PKCα with two different shRNAs inhibit viability of M-HCC cells as assessed by a colony formation assay. The decrease in colony number is significant (*p* < 0.01) in all cases. **e** PKC inhibition by 100 nM UCN-01 significantly reduced (SNU475) or completely inhibited (SNU387, SKHep1) the hepatosphere forming ability of M-HCC cell lines. PLC cells overexpressing ZEB1 (ZEB1-induced EMT) also showed a strong inhibition of hepatosphere formation ability upon UCN-01 treatment (bars on the right). The decrease in hepatosphere number was significant (*p* < 0.05) in all cases

As PKC pathway is a well-documented inducer of tumorigenesis^24,25^, its activation is reported during EMT-associated CSC formation^7^ and we observed low nM *IC50* values of UCN-01 during viability tests (Fig. 5), we investigated PKC activity and PKC family expression in HCC. Among all PKC isoforms, only PKCα abundance correlated with mesenchymal status (Fig. 6a). Other PKC isoforms were either undetectable (data not shown), expressed equally (PKCβ) or showed no correlation with the EMT (PKCβ, δ, ε, θ). A specific PKCα substrate or pan-PKC-substrate antibodies showed strong signal only in M-HCC cells suggesting PKCα is the candidate kinase that is inhibited by PKC inhibitors (Fig. 6a)^26,27^. To assess PKCα and ZEB1 expression in a clinical context we explored a HCC cohort (LIHC) of TCGA database. *ZEB1* and *PRKCA* mRNA expressions showed a significant (*p* = 2.2 × 10^−16^) and positive (*r* = 0.479) association, similar to that of *ZEB1* and *Vimentin* (*p* = 3.04 × 10^−8^, *r* = 0.308, Supplementary Fig. S5), which confirms M-HCC specific expression of PKCα in human samples.

These results prompted us to investigate the comparative effect of UCN-01 and Midostaurin on PKC activity in Hepatoma. We found that as little as 20 nM of UCN-01 was capable of inhibiting PKC activity better than 2 μM Midostaurin (Fig. 6b). This significant difference explains higher *IC50* values and delayed apoptosis for Midostaurin (36 vs 8 h, Fig. 5, Supplementary Fig. S3). To determine whether PKCα is a critical regulator of M-HCC cell viability, we knocked-down *PRKCA* using two different shRNAs. Short-term *PRKCA* downregulation induced phospho-PKC-substrate signal reduction validating that it is the active PKC family member in M-HCC cells (Fig. 6c). Despite significant endeavours, we were unable to create stable *PRKCA* knockdown M-HCC cells but obtained clones from the same lentiviral backbone suggesting that the observed effect of PKCα deficiency is genuine (data not shown). A colony formation assay using three M-HCC cell lines and two *PRCKA* targeting shRNAs revealed cell survival is significantly reduced upon prolonged *PRKCA* downregulation (Fig. 6d). Similar to genetic targeting of *PRKCA*, low-dose UCN-01 treatment inhibited colony forming ability of Hepatoma cell line panel but the observed effect was total repression in M-HCC cells (Supplementary Fig. 3C). Importantly, treating M-HCC or PLC/PRF/5 cells overexpressing ZEB1 during hepatosphere formation with 100 nM UCN-01 resulted in a major decrease in sphere/cluster formation (Fig. 6e). These results are in concordance with a previous study^28^ and suggest that M-HCC cells are critically dependent on PKCα for survival.

**Fig. 7:**
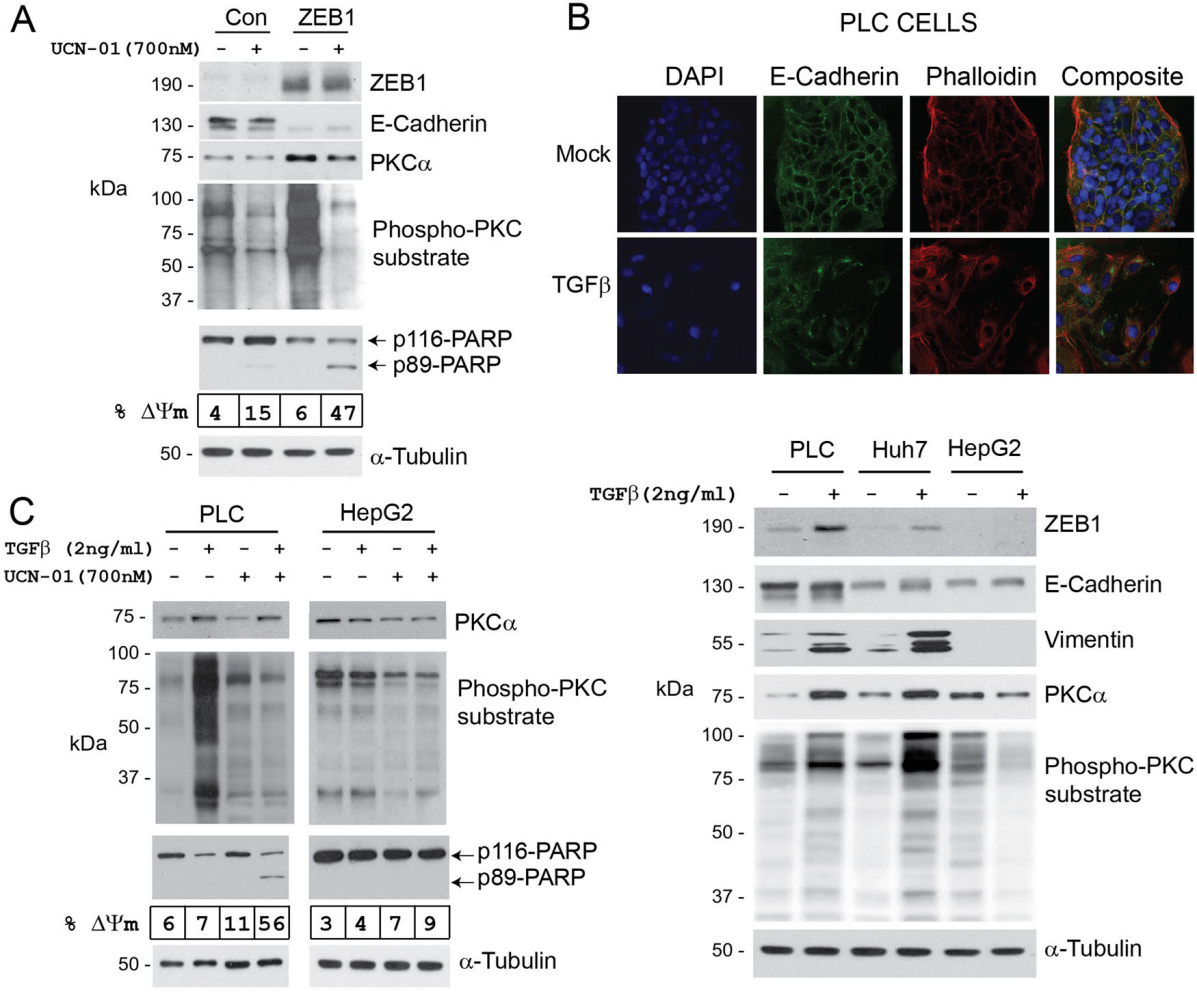
ZEB1- or TGFβ-induced EMT renders E-HCC cells sensitive to UCN-01. **a** PLC/PRF/5 cells were transfected with pCDNA4 (Con) or pCDNA4-ZEB1 (ZEB1) plasmids. Seventy two hours later and after hallmarks of EMT were observed, cells were treated with 700 nM UCN-01 for 8 h. UCN-01 treatment resulted in significant (47%) apoptosis in ZEB1-expressing cells compared with the control (15%) as assessed by PARP cleavage and mitochondria depolarization. PKCα protein abundance and PKC activity were increased as a result of ZEB1-induced EMT. **b** Three E-HCC cell lines were treated with TGFβ (2 ng/ml) for 72 h. EMT was confirmed by the analysis of cell morphology, E-Cadherin and vimentin protein expression and localization. All E-HCC cells, with the exception of HepG2, responded to TGFβ showing increased ZEB1 and vimentin expression, cytoplasmic re-distribution of E-Cadherin, disappearance of cortical actin (phalloidin staining) and cell scattering. PKCα abundance and activity were also increased as a result of TGFβ-induced EMT. **c** TGFβ-responsive (PLC/PRF/5) and non-responsive (HepG2) cells were incubated with TGFβ for 72 h and treated with 700 nM UCN-01 for an additional 8 h. PKC abundance and activity were assessed by western blotting along with the pro-apoptotic activity of UCN-01 as detected using flow cytometry (mitochondria depolarization, % ΔΨm) and PARP cleavage. TGFβ treatment rendered PLC/PRF/5, but not HepG2, cells sensitive to UCN-01-induced cell death

To exclude genetic variations in Hepatoma cells as the cause of selective sensitivity to UCN-01, we analysed ZEB1- and TGFβ-induced EMT models. ZEB1-induced EMT strongly increased PKCα expression as well as PKC-substrate phosphorylation (Fig. 7a). ZEB1-induced EMT also sensitised PLC/PRF/5 cells to UCN-01-induced apoptosis (Fig. 7a). TGFβ is a well-documented inducer of EMT in HCC^29^. As reported previously, PLC/PRF/5 and Huh7, but not HepG2, cells responded to TGFβ, displaying hallmarks of EMT such as cell scattering, increased expression of ZEB1 and vimentin, downregulation of E-cadherin and formation of cortical actin (Fig. 7b)^30^. TGFβ also induced the expression of PKCα and PKC activity in PLC/PRF/5 and Huh7 cells (Fig. 7b) but not in HepG2. Thus, we chose PLC/PRF/5 (TGFβ-responsive) and HepG2 (TGFβ-non-responsive) cells to investigate the action of UCN-01 following TGFβ treatment. Activation of TGFβ-induced EMT increased PKCα abundance (Fig. 7b) and sensitised PLC/PRF/5 cells to UCN-01-mediated apoptosis, as assessed by PARP cleavage and mitochondria depolarization, together with inhibition of PKC activity (Fig. 7c). HepG2 cells did not upregulate PKCα or responded to UCN-01 (Fig. 7b, c).

**Fig. 8:**
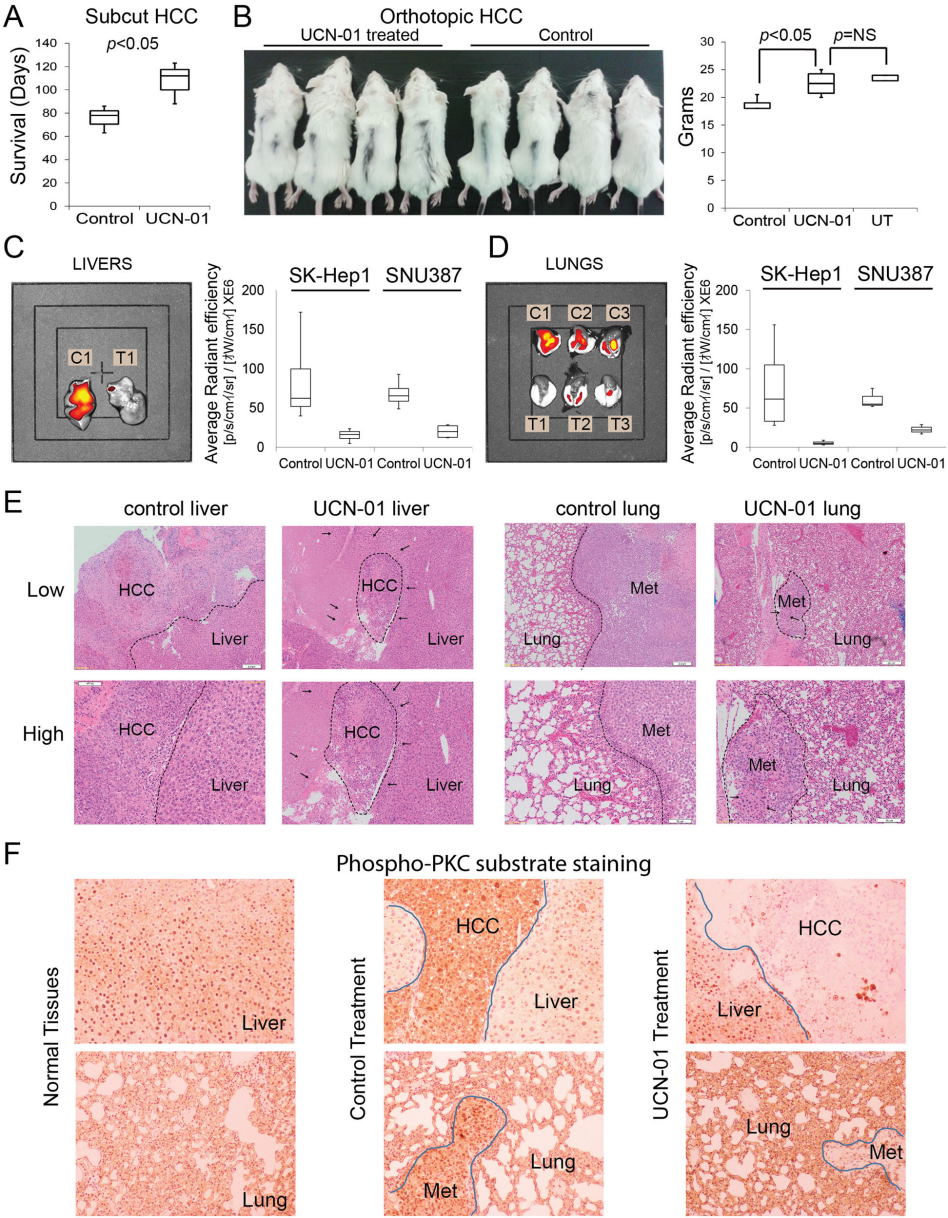
UCN-01 has in vivo efficacy for metastatic HCC. **a** To test the toxicity and tolerability of UCN-01, SKHep1 cells were subcutaneous injected to SCID BALB/C mice (10^5^ cells). UCN-01 was administered at a weekly dose of 2 mg/kg for 14 weeks after tumours became palpable. UCN-01 reduced tumour growth and therefore animals survived longer meeting welfare criteria. **b** SNU387 or SKHep1 cells were injected orthotopically (2.5 × 10^5^ cells, mixed 50/50 with Matrigel, as 60 μl) to the liver parenchyma of SCID BALB/C mice. UCN-01 was administered at weekly intervals after a 10-day healing time. Three weeks after injection, cachexia secondary to weight loss became evident in the control group. The experiment was terminated when weight loss exceeded 20% compared with untreated group. Livers (**c**) and lungs (**d**) were analysed for the presence of cancer cells using 800CW-2DG probe. UCN-01 treated animals (T1–T3) showed reduced fluorescence compared with control group (C1–C3). **e** Histopathological analysis revealed poorly differentiated HCC in livers and lungs as presented in ×40 (low) and ×100 (high) magnifications. The tumour boundaries were marked with dashed lines. In all cases UCN-01 treatment reduced tumour burden significantly (*p* < 0.05). The arrows are marking the areas of condensed nucleus (Pyknosis) in UCN-01 treated samples marking dead cancer cells. **f** IHC using phospho-PKC-substrate antibody revealed that normal livers and lungs have marginally low PKC activity (left panels). Primary and secondary HCC cells have significantly higher PKC-substrate phosphorylation (middle panels) and UCN-01 treatment eliminated PKC activity (right panels)

Finally, to investigate the in vivo efficacy and tolerability of UCN-01, SKHep1 wells (M-HCC) were injected subcutaneously to SCID mice. UCN-01 administration prolonged survival significantly to an extent that the final animal in the treated group was culled without developing a significant tumour (Fig. 8a). UCN-01 was well tolerated at a dose of 2 mg/kg/week. Observing the anti-tumour activity of UCN-01 in a M-HCC cell line prompted us to investigate whether it impacts tumorigenicity of E-HCC cells. All three E-HCC cell lines (Huh7, PLC/PRF/5 and HepG2) were tested in subcutaneous context. The tumours exposed to UCN-01 were smaller (up to half weight) compared with controls (Supplementary Fig. 6). These results suggest UCN-01 has anti-tumour activity for all Hepatoma cell lines but more potent in eliminating M-HCC cells.

To study the efficacy of UCN-01 in treating metastatic HCC in a physiological micro-environment, we developed orthotopic murine models with SNU387 and SKHep1 cells and followed tumour growth and metastasis with a fluorescent glucose probe (800CW-2DG). Treatment, delivery and analysis of data were conducted in an investigator-blinded manner. Orthotopic tumours formed more quickly as control-treated animals showed cachexia within 3 weeks (Fig. 8b, left panel). UCN-01 treatment did not result in significant weight loss as compared with wild-type mice (Fig. 8b, right panel). When experiment was terminated due to extensive cachexia, livers and lungs were analysed using in vivo imager. The fluorescence, marking the presence of cancer cells, emitted from livers of UCN-01 reduced significantly compared with control animals (Fig. 8c) indicating that UCN-01 inhibited the survival of M-HCC cells. Notably, the fluorescence from dissected lungs was also significantly lower in the UCN-01 group compared with control animals (Fig. 8d), indicating both M-HCC cell lines are truly metastatic and the anti-neoplastic action of UCN-01 takes place in the main- and end-organs of interest. Histopathological analysis confirmed the presence of Hepatoma in livers and lungs of the animals paralleling the fluorescence emission (Fig. 8e). The tumours obtained from UCN-01 treated animals had less PKC activity (Fig. 8f). We also observed areas of pyknotic and shrunken nuclei indicating tumour cell death from UCN-01 (Fig. 8e). Careful examination of non-tumour parts of UCN-01 treated lungs and livers showed no pathological evidence of injury to non-neoplastic cells, marking the selectivity of UCN-01 to only M-HCC cells and confirming our in vitro observations. These results indicate that UCN-01 is tolerable for epithelial cells, and can effectively induce apoptosis in M-HCC cells at primary and clinically relevant secondary sites.

## Discussion

Despite significant advances in surgery and chemotherapy, metastasis and therapy-resistance are key factors contributing to HCC-related mortality^3^. Consequently, the identification of surrogate biomarkers for micrometastases, and the development of targeted therapeutics are key areas of unmet need in HCC.

In this study we performed an empirical expression analysis of ZEB family proteins and identified ZEB1 as an independent and externally validated biomarker of oncological outcome in HCC. We characterised its expression in a panel of commonly used Hepatoma cell lines, and demonstrated that ZEB1-induced EMT leads to chemoresistance to conventional chemotherapeutic agents. ZEB1 is able to stratify HCC into epithelial and mesenchymal sub-types both in vitro and in vivo. We demonstrated for the first time that PKC pathway is activated in mesenchymal HCC cells and that contributes to their survival. We identified UCN-01 having selective activity to M-HCC cells in vitro and in vivo, in doses that can be extrapolated to man, and without effect on the non-malignant cells, at least in mice.

Previous studies showed the expression EMT-TFs to predict for OS and DFS in HCC patients^31–33^. Our study is the first, however, to use patient material from multiple independent HCC cohorts and follow biomarker reporting guidelines. Adequately powered prospective randomised-trials are necessary to fully confirm ZEB1 as a biomarker with clinical utility in HCC.

Metastasis-chemoresistance association is a major challenge in cancer research. We previously demonstrated that ZEB2-induced EMT blocks DNA damage-induced initiation of intrinsic apoptosis machinery^11^. A recent study marked the importance of EMT in cancer chemoresistance using genetically traceable models^34^. These results are in concordance with our findings since M-HCC cells tolerated DNA damaging agents better than E-HCC cells. Sorafenib was proven to have clinical utility in HCC treatment but, as shown in this study and others, there is no evidence that it can selectively target M-HCC cells^35^. Our results also support earlier findings that activation of EMT by ZEB1 or TGFβ overrides Ras, and therefore RTK addiction^8^. Therefore, targeting metastatic carcinoma cells with RTK inhibitors or through Ras pathway (such as with PDFG-R/Raf inhibitor Sorafenib) may not yield significant clinical benefit in metastatic HCC.

Here we also showed that ZEB1-expressing, mesenchymal and chemoresistant (M-HCC) cells became addicted to PKCα, and can be selectively eliminated by PKC inhibitors. These compounds are derived from a relatively non-specific kinase inhibitor, staurosporine. UCN-01 is hydroxylated-staurosporine which restricted its activity to kinases with a free water molecule in their ATP binding pocket, such PKC, PDK1 and CHK1^22,36,37^. Like other inhibitors, UCN-01 becomes more specific when used at a lower concentrations. Considering the low nM *IC50* values of UCN-01 (<62 nM in M-HCC cells), it is feasible to assume its activity to be specific rather than broad. The fact that genetic modelling of PKCα inactivation, as shown in this study and elsewhere^28^, also reduced HCC viability supports our assumption. However, we cannot exclude the possibility that additional pro-survival kinases such as PDK1 or AKT are inhibited by UCN-01 in assays where we used higher concentrations (100–700 nM) and looked at apoptosis in the short term (8 h). Importantly, UCN-01 induced very little/no apoptosis in E-HCC cells or fibroblasts at these conditions. It is also noteworthy to say that UCN-01 showed partial but significant efficacy also in E-HCC cells in long-term treatments such as colony formation assays or in vivo experiments (Supplementary Figs. S3C and S6). This could be due to the fact that all E-HCC cells contain a small proportion of CSCs and UCN-01 is very potent in killing this population. Also, when injected to animals, E-HCC cells are exposed to EMT inducing soluble molecules such as TGFβ or chemokines. Overall, UCN-01 showed greater efficacy in killing M-HCC cells, however, the fact that it is inhibiting the viability of E-HCC cells in long-term assays is another benefit to justify its use in HCC patients.

A recent study investigating pro-survival pathways in metastatic colonization suggested inhibition of PKC signalling by UCN-01 significantly decreased cancer metastasis in vivo by inducing apoptosis^38^. Similarly, overexpression of PKCα and selective activation of PKC signalling in mesenchymal-BC cells and BC stem cells was described^7^. BC CSCs become sensitive to PKC inhibitors which are also stausporine derivatives. Of note, these inhibitors have never been tested in man, whereas UCN-01 has been assessed in 22 phase I/II human clinical trials showing clinical benefit in progression-free survival. However, lack of biomarker-driven patient stratification has impeded its progression.

In recent years, identification of rare populations (e.g. CD133, CD90 or EpCAM positive) from mainly AFP + HCC (E-HCC) cells and assessing their CSC features has been an approach in attempts to identify HCC stem cells^16^. Unlike other cancers, activatable models of EMT in HCC, such as used in this study, has not been utilised. Our findings supported by data from independent public databases (CCLE and Genentech) suggest that CD44^high^/CD24^low^ phenotype is associated with ZEB1-induced EMT (Fig. 4c) and anoikis resistance which are properties of M-HCC cells (Fig. 4a, b); therefore they should be considered for markers of HCC stem cells. Several studies reported CD44^high^ HCC cells having a greater CSC capacity, without, however, considering their EMT status^19,39–41^. As few as 10 SKHep1 cells were shown to induce metastatic tumours in 27 of 28 animals injected showing their CSC capacity in vivo^39^. Forced or endogenous ZEB1 expression resulted in enhanced hepatosphere formation suggesting EMT is critical for CSC attributes for HCC.

EMT related genetic/epigenetic changes were proposed to drive the formation of differentiated (epithelial) or undifferentiated (mesenchymal) subclasses of primary and secondary tumours^4,42^. The survival and response to chemotherapy significantly differs in these types of disease; and patients with an undifferentiated phenotype have significantly poorer prognosis^42^. The considerable complexity of cancer progression requires a therapy where combinations of agents targeting epithelial (Doxorubicin) and mesenchymal (UCN-01) subpopulations of HCC is required. We propose stratifying HCC patients according to ZEB1 expression, and treating patients with ZEB1-positive tumours with an UCN-01 containing combination therapy for a successful treatment.

## Supplementary information

supplementary documents.

## References

[1] El-Serag HB (2011). Hepatocellular carcinoma. New Engl. J. Med..

[2] Asghar U, Meyer T (2012). Are there opportunities for chemotherapy in the treatment of hepatocellular cancer?. J. Hepatol..

[3] Forner A, Llovet JM, Bruix J (2012). Hepatocellular carcinoma. Lancet.

[4] Valastyan S, Weinberg RA (2011). Tumor metastasis: molecular insights and evolving paradigms. Cell.

[5] Singh A, Settleman J (2010). EMT, cancer stem cells and drug resistance: an emerging axis of evil in the war on cancer. Oncogene.

[6] Thiery JP, Acloque H, Huang RY, Nieto MA (2009). Epithelial-mesenchymal transitions in development and disease. Cell.

[7] Tam WL (2013). Protein kinase C alpha is a central signaling node and therapeutic target for breast cancer stem cells. Cancer Cell.

[8] Singh A (2009). A gene expression signature associated with “K-Ras addiction” reveals regulators of EMT and tumor cell survival. Cancer Cell.

[9] McShane LM (2005). Reporting recommendations for tumor marker prognostic studies. J. Clin. Oncol..

[10] Moore HM (2011). Biospecimen reporting for improved study quality (BRISQ). J. Proteome Res..

[11] Sayan AE (2009). SIP1 protein protects cells from DNA damage-induced apoptosis and has independent prognostic value in bladder cancer. Proc. Natl Acad. Sci. USA.

[12] Sayan AE, Sayan BS, Findikli N, Ozturk M (2001). Acquired expression of transcriptionally active p73 in hepatocellular carcinoma cells. Oncogene.

[13] Mejlvang J (2007). Direct repression of cyclin D1 by SIP1 attenuates cell cycle progression in cells undergoing an epithelial mesenchymal transition. Mol. Biol. Cell.

[14] Llovet JM (2008). Sorafenib in advanced hepatocellular carcinoma. New Engl. J. Med..

[15] Kalluri R, Weinberg RA (2009). The basics of epithelial-mesenchymal transition. J. Clin. Investig..

[16] Yamashita T, Wang XW (2013). Cancer stem cells in the development of liver cancer. J. Clin. Investig..

[17] Mani SA (2008). The epithelial-mesenchymal transition generates cells with properties of stem cells. Cell.

[18] Petryszak R (2016). Expression Atlas update-an integrated database of gene and protein expression in humans, animals and plants. Nucleic Acids Res..

[19] Yamashita T (2013). Discrete nature of EpCAM+ and CD90+ cancer stem cells in human hepatocellular carcinoma. Hepatology.

[20] Chaffer CL, San Juan BP, Lim E, Weinberg RA (2016). EMT, cell plasticity and metastasis. Cancer Metastasis Rev..

[21] Peinado H, Olmeda D, Cano A (2007). Snail, Zeb and bHLH factors in tumour progression: an alliance against the epithelial phenotype?. Nat. Rev. Cancer.

[22] Fabian MA (2005). A small molecule-kinase interaction map for clinical kinase inhibitors. Nat. Biotechnol..

[23] Goekjian PG, Jirousek MR (2001). Protein kinase C inhibitors as novel anticancer drugs. Expert Opin. Investigational Drugs.

[24] Castagna M (1982). Direct activation of calcium-activated, phospholipid-dependent protein kinase by tumor-promoting phorbol esters. J. Biol. Chem..

[25] Griner EM, Kazanietz MG (2007). Protein kinase C and other diacylglycerol effectors in cancer. Nat. Rev. Cancer.

[26] Araujo H, Danziger N, Cordier J, Glowinski J, Chneiweiss H (1993). Characterization of PEA-15, a major substrate for protein kinase C in astrocytes. J. Biol. Chem..

[27] Nishikawa K, Toker A, Johannes FJ, Songyang Z, Cantley LC (1997). Determination of the specific substrate sequence motifs of protein kinase C isozymes. J. Biol. Chem..

[28] Lin SB (2000). In vitro and in vivo suppression of growth of rat liver epithelial tumor cells by antisense oligonucleotide against protein kinase C-alpha. J. Hepatol..

[29] Reichl P, Haider C, Grubinger M, Mikulits W (2012). TGF-beta in epithelial to mesenchymal transition and metastasis of liver carcinoma. Curr. Pharm. Des..

[30] Senturk S (2010). Transforming growth factor-beta induces senescence in hepatocellular carcinoma cells and inhibits tumor growth. Hepatology.

[31] Lee TK (2006). Twist overexpression correlates with hepatocellular carcinoma metastasis through induction of epithelial-mesenchymal transition. Clin. Cancer Res..

[32] Yang MH (2009). Comprehensive analysis of the independent effect of twist and snail in promoting metastasis of hepatocellular carcinoma. Hepatology.

[33] Zhou YM (2012). Clinicopathological significance of ZEB1 protein in patients with hepatocellular carcinoma. Ann. Surg. Oncol..

[34] Fischer KR (2015). Epithelial-to-mesenchymal transition is not required for lung metastasis but contributes to chemoresistance. Nature.

[35] van Zijl F (2011). A human model of epithelial to mesenchymal transition to monitor drug efficacy in hepatocellular carcinoma progression. Mol. Cancer Ther..

[36] Davies SP, Reddy H, Caivano M, Cohen P (2000). Specificity and mechanism of action of some commonly used protein kinase inhibitors. Biochem J..

[37] Komander D (2003). Structural basis for UCN-01 (7-hydroxystaurosporine) specificity and PDK1 (3-phosphoinositide-dependent protein kinase-1) inhibition. Biochem J..

[38] Hong SH, Ren L, Mendoza A, Eleswarapu A, Khanna C (2012). Apoptosis resistance and PKC signaling: distinguishing features of high and low metastatic cells. Neoplasia.

[39] Eun JR (2014). Hepatoma SK Hep-1 cells exhibit characteristics of oncogenic mesenchymal stem cells with highly metastatic capacity. PLoS ONE.

[40] Hashimoto N (2014). Cancer stem-like sphere cells induced from de-differentiated hepatocellular carcinoma-derived cell lines possess the resistance to anti-cancer drugs. BMC Cancer.

[41] Chen X (2011). Epithelial mesenchymal transition and hedgehog signaling activation are associated with chemoresistance and invasion of hepatoma subpopulations. J. Hepatol..

[42] Brabletz T (2012). To differentiate or not-routes towards metastasis. Nat. Rev. Cancer.

