# Supplementary material for: Protein kinase C inhibitors override ZEB1-induced chemoresistance in HCC
Source: Cell Death Dis. 2019 Sep 23;10(10):703. doi: 10.1038/s41419-019-1885-6 (PMC6755133; doi:10.1038/s41419-019-1885-6)
Supplement: Supplementary file 1 — supplementary documents. [file 41419_2019_1885_MOESM1_ESM.docx]

**SUPPORTING INFORMATION**

Protein Kinase C inhibitors override ZEB1 induced chemoresistance in HCC

Rahul Sreekumar^1,2^, Muhammad Emaduddin^1^, Hajir Al-Saihati^1^, Karwan Moutasim^1^, James Chan^1^, Marcello Spampinato^3^, Rahul Bhome^1,2^, Ho Ming Yuen^4^, Claudia Mescoli^5^, Alessandro Vitale^6^, Umberto Cillo^6^, Massimo Rugge^5^, John Primrose^1,2^, Mohammad Abu Hilal^2^, Stephen Thirdborough^1^, Eugene Tulchinsky^7, 8, 9^, Gareth Thomas^1^, Alex Mirnezami^1,2^ and A. Emre Sayan^1^

1- Supplementary Materials and Methods.

2- Supplementary table 1.

3- Supplementary table 2.

4- Supplementary table 3.

5- Supplementary Figure 1

6- Supplementary Figure 2

7- Supplementary Figure 3

8- Supplementary Figure 4

9- Supplementary Figure 5

10-Supplementary Figure 6

11- Supplementary Figure 7

12- Supplementary References

**Supplementary Materials and Methods**

***Patient material and analysis of ZEB immuno expression***

Paraffin embedded tissue sections (from human or mouse) were placed on microscope slides (SUPERFROST plus, Menzel-Glaser, Braunschweig, Germany) and baked overnight at 37°C. Sections were deparaffinised in Xylene, rehydrated in graded concentrations of ethanol and endogenous peroxidase activity was inhibited using 0.5% hydrogen peroxide. Antigen retrieval was done by microwave boiling of tissues for 15 minutes in 0.01M citrate buffer (pH: 6.0). Non-specific binding was blocked using Avidin/biotin blocking kit (Vector Laboratories, CA, USA), and slides were incubated overnight at 4°C with primary antibody; rabbit anti-ZEB1 (H102, Santa Cruz Biotech, 1:400 dilution), mouse anti-E-Cadherin (Dako, Clone NCH-38, 1:500 dilution), anti-PKC substrate (Cell Signaling, 1:1000) and rabbit anti-ZEB2 (CUK2, home-made polyclonal antibody raised against the N-terminal 360 amino acids of human ZEB2, 1:750) (1, 2). After washing with phosphate buffer saline (PBS), sections were incubated with secondary antibody, streptavidin biotin-peroxidase complexes and primary antibody detected using Vector DAB peroxidase substrate kit (Vector Laboratories, CA, USA). Sections were counterstained in haematoxylin, dehydrated in ethanol and mounted. Two pathologists performed scoring independently and a joint re-assessment was done if a discrepancy existed.

***Assessment of cell viability, apoptosis and motility***

To observe effects of various drugs on cell viability, a microscopy-based system was used. Briefly, 10000 cells were seeded in 48 well plates and incubated with drugs (6 concentrations in quadruplicate for *IC*50 assay). Eight hours later, wells were washed and fresh media was added. After 48-96 hours, cells were washed with PBS, fixed with ice cold acetone/methanol (50/50) and stained with DAPI (Molecular Probes). A semi-automated system was used to take pictures from the centre of the each well (UV channel, 40X magnification, Olympus-CKX41) and the cell number was determined using ImageJ program as described before (3). Intact nuclei are counted as “live cells”. Apoptosis was quantitated using flow-cytometry, by assessing mitochondria depolarization (TMRE) or sub-G1 cellular DNA content as described previously (4). Additionally, PARP cleavage was always assessed as a second technique to observe and validate the biochemical hallmarks of apoptosis. Crystal violet assay or colony formation assay was performed similar to viability assays. Briefly, cells were exposed to drugs (or solvent) for 6-8 hours, washed and fresh media added. Four days later (or when the control cells reached 100% confluency), cells were washed, fixed with acetone/methanol (50/50) and stained with crystal violet. Representative low magnification pictures were taken. For colony formation assay, 500 cells were seeded in 6cm dishes in triplicate, treated with drugs and fixed-stained with crystal violet. Counting of colonies was done manually by 2 indeendent sceientists and averaged. Cell motility was analysed using Transwell membrane inserts (8μm, BD Biosciences) in 24 well tissue culture plates as previously described (3). Caspase3/7 assay was purchased from Promega (Caspase-Glo® 3/7) and performed as instructed by supplier.

***Expression analysis, Western Blotting and Immunofluorescence***

For immunofluorescence, cells were seeded on glass coverslips and stained with relevant primary antibodies as previously described (1). To detect EMT related actin-cytoskeleton changes Alexa-568 conjugated phalloidin (Molecular Probes) was used. Western blotting was performed as described previously (5). The primary antibodies used are: PARP, Vimentin, pan-PKC substrate, PEA-15, S104 PEA-15, (Cell Signalling), ZEB1, actin (Santa Cruz), E-Cadherin and all PKC antibodies (BD), α-Tubulin and HA-tag (Sigma), ZEB2/SIP1 (home made, described in (1)). The membranes were incubated with HRP-conjugated secondary antibodies and the signal was revealed using West-Dura substrate (Pierce) and autoradiography films. RNA extraction and cDNA synthesis have been performed as described before (5). The validated assays for qPCR has been purchased from Qiagen (CD133 (*PROM*): QT02394343, CD90 (*THY1*): QT00023569, *CD44*: QT00073549, *CD24*: QT01871044, *EpCAM*: QT00000371). Syber green has been used in qPCR reaction and beta-actin (*ACTB*): QT00095431) probe was been used for normalization.

***Hepatosphere formation assay***

Initially, cells were suspended and washed with DMEM/F12 media twice. Then, cells were counted and diluted to yield 100 cells/100ul in sphere induction medium (DMEM/F12, 0.5% glucose, 1X ITS-G reagent containing insulin, transferrin, selenium (Thermo-Fisher), 1X antibiotic solution (penicilin/streptomycin), 0.5% BSA, B27 reagent (1/50 dilution, Thermo-Fisher), 10ng/ml recombinant EGF (Peprotech), 10ng/ml recombinant bFGF (Peprotech) and 0.25μM progesterone (Sigma). The cells were seeded as 100μl (100 cells) to 6 wells of a 96 well plate coated with poly-HEMA. The cells were kept in an incubator with humidified atmosphere and 5% CO_2_ for 6 days. Shaking or moving plates were avoided during this period. After that, the cell clusters larger than 50μm were counted as hepatospheres.

***Bioinformatic analysis***

Raw read counts from a HCC dataset (LIHC) were imported from TCGA into edgeR (6) and normalized using the limma-voom algorithm (7). Linear regression analysis of normalized log-cpm data was carried out in Graph Pad Prism v6. Non-hierarchial clustering was performed using loading raw expression data from databases (Geneatlas) or ΔΔct values from qPCR analysis to R software and using Pheatmap add-on.

**Supplementary table 1:** Association between the expression of E-Cadherin, ZEB1, ZEB2(SIP1) and clinical/ pathological parameters in the UK cohort.

**Supplementary table 2**: Association between the expression of ZEB1 and clinical/ pathological parameters in the Italian cohort. Please note that some patients from Italian cohort were omitted from statistical analysis if relative data was not available. This is indicated in detail in Supplementary table 3.

**Supplementary table 3:** Pre-analytical biospecimen factors in compliance with BRISQ tier one reporting guidance, and REMARK profile for patient cohorts, variables evaluated, and statistical analyses conducted.

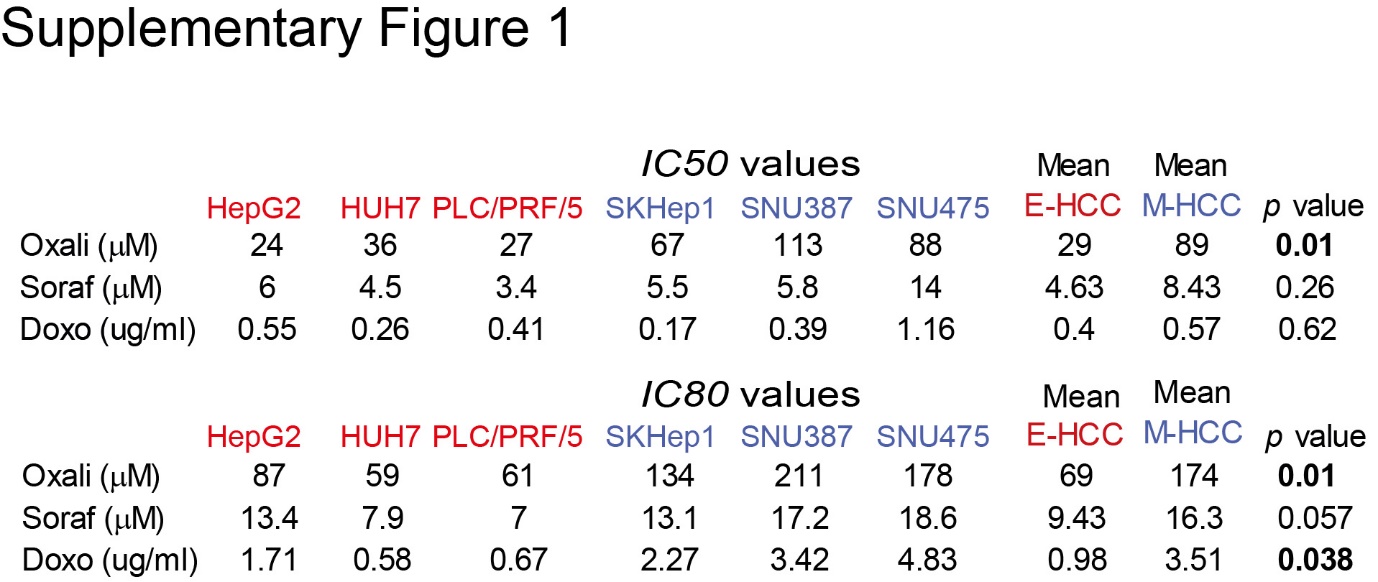


**Supplementary Figure 1:** A panel of 6 hepatoma representing 3 E-HCC and 3 M-HCC cell lines were subjected to viability analysis with clinically relevant drugs used in HCC treatment. *IC50* (top) and *IC80* (bottom) values were identified for each cell line and also the mean *IC* values were calculated for E-HCC and M-HCC groups. Sorafenib did not stratify E- and M-HCC groups at *IC50* or *IC80*. Oxaliplatin showed significant difference at both *IC50* and *IC80*. Doxorubicin only stratified these 2 group of cell lines at *IC80*.


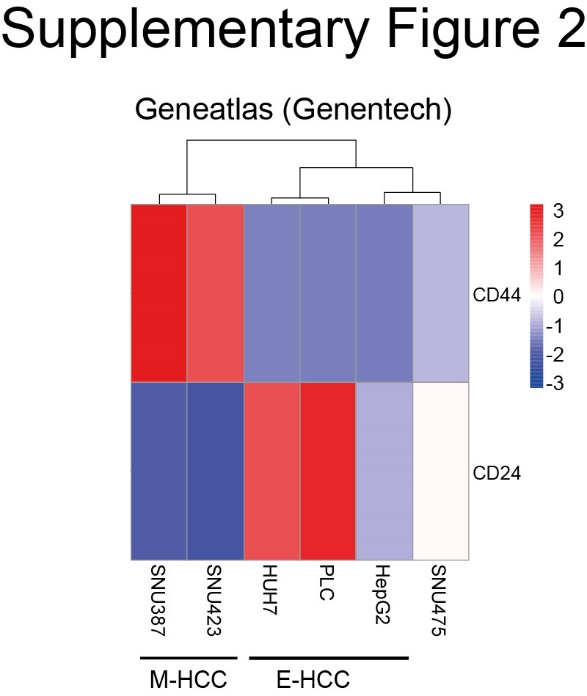


**Supplementary Figure 2:** CD44 and CD24 RNA expression stratifies M-HCC and E-HCC cells in Genentech cohort of Geneatlas database with the exception of SNU475.


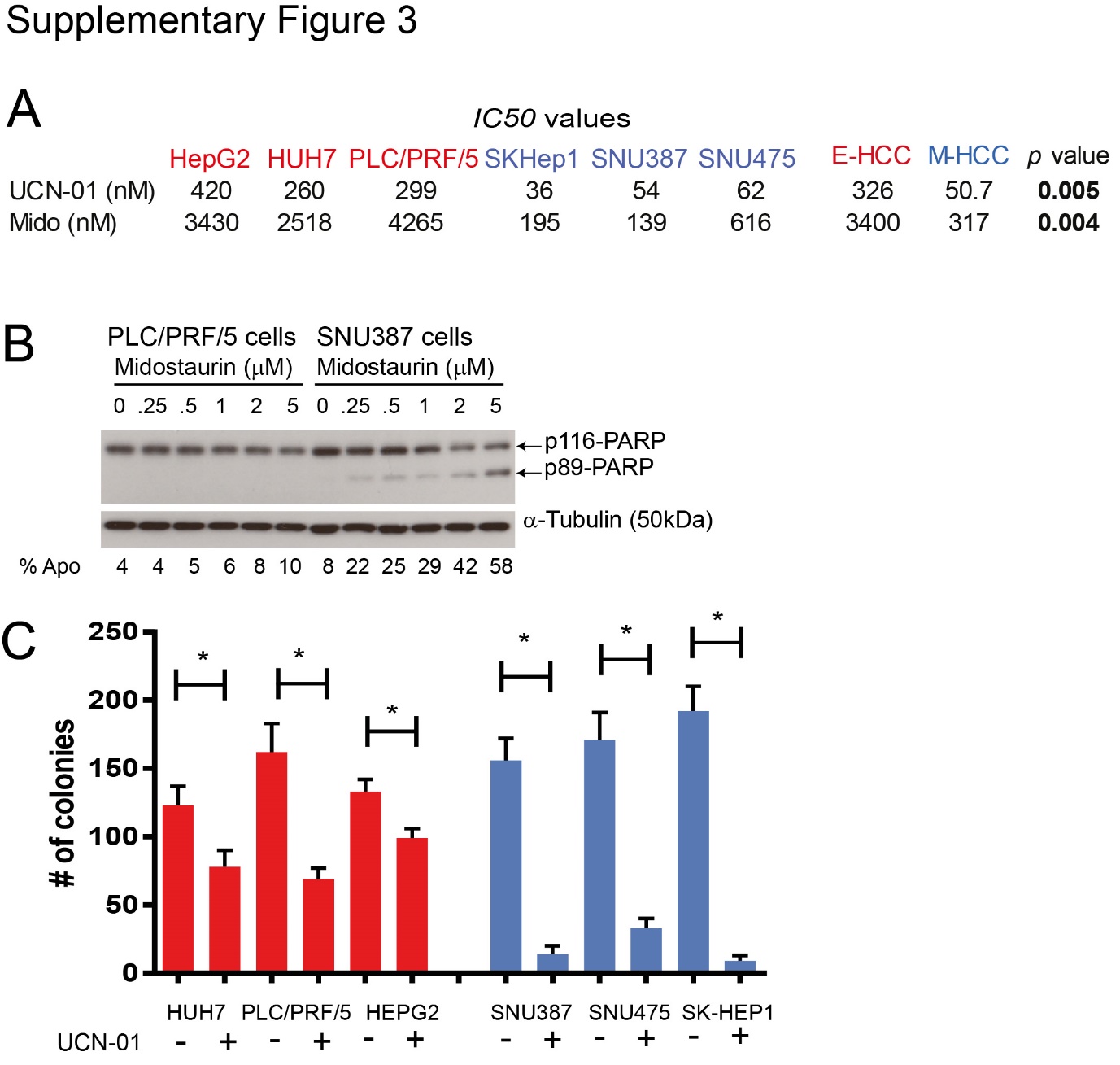


**Supplementary Figure 3: (A)** The panel of 6 Hepatoma cell lines representing epithelial and mesenchymal characteristics were subjected to viability assay upon UCN-01 or Midostaurin treatment and *IC50* values were calculated for individual cell lines and by morphological clustergin (mean *IC50* of E- and M-HCC cells). Both PKC inhibitors stratified HCC cells according to their morphological status at their *IC50*. **(B)** Long term treatment (36h) of M-HCC cells was necessary to observe detectable apoptosis (indicated by the presence of p89 PARP). At this condition midostaurin showed no pro-apoptotic activity in E-HCC cells such as PLC/PRF/5. **(C)** The HCC cell line panel comprised of 3 E- and 3M-HCC cell lines were subjected to colony formation assay using 100nM UCN-01. The drug repressed colony formation of both cell types but the decrease is more prominent in M-HCC cells (more than 80%) as compared to E-HCC (halved of decreased less).


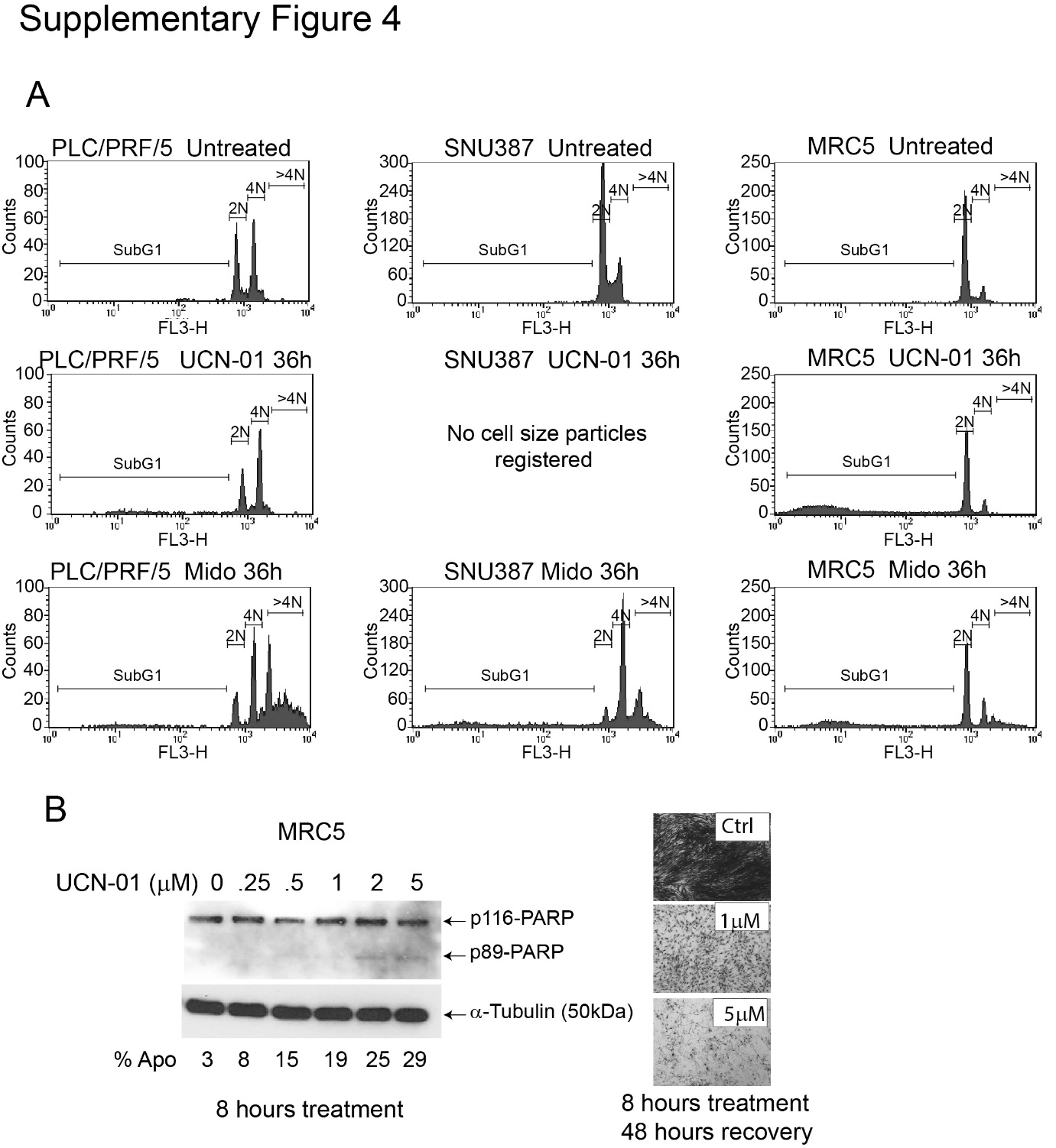


**Supplementary Figure 4:** Morphologically different Hepatoma cells respond differently to PKC inhibitors. **(A)** E-HCC (PLC/PRF/5), M-HCC (SNU387) and MRC5 (human fibroblast) cells were incubated with 1μM UCN-01 (middle panels) and 5μM Midostaurin (lower panels) for 36 hours (continous incubation). DNA content analysis of these cells revealed that UCN-01 had minor or mediocre effect, as defined by an increase in subG1 population, in PLC cells or fibroblasts respectively, as compared to control (top panels) but no viable cell was present in SNU387 sample. The cells obtained from this sample registered less than 1% cell-sized particles due to extensive apoptosis. Continuous exposure to Midostaurin induced minor apoptosis in all cells tested accompanied by a strong polyploidy response (>4N cell population, bottom panels, indicated by >4n). **(B)** Non-malignant mesenchymal cells, such as MRC5 human lung fibroblasts, are more sensitive to UCN-01 than epithelial but less than mesenchymal HCC cells. Biochemical hallmarks of apoptosis (8 hour continuous treatment, left panel) and recovery after treatment (8 hours treatment followed by 48 hours recovery, right panel) were analysed. Unlike E-HCC cells, higher concentrations of UCN-01 (>1μM) induced detectable PARP cleavage in MRC5 fibroblasts. Crystal violet (recovery) assay showed that UCN-01 was cytostatic/toxic to MRC5 fibroblasts, however, there was a reasonable amount of live cells after exposure to 1μM UCN-01. This concentration of UCN-01 induced complete elimination of M-HCC cells.


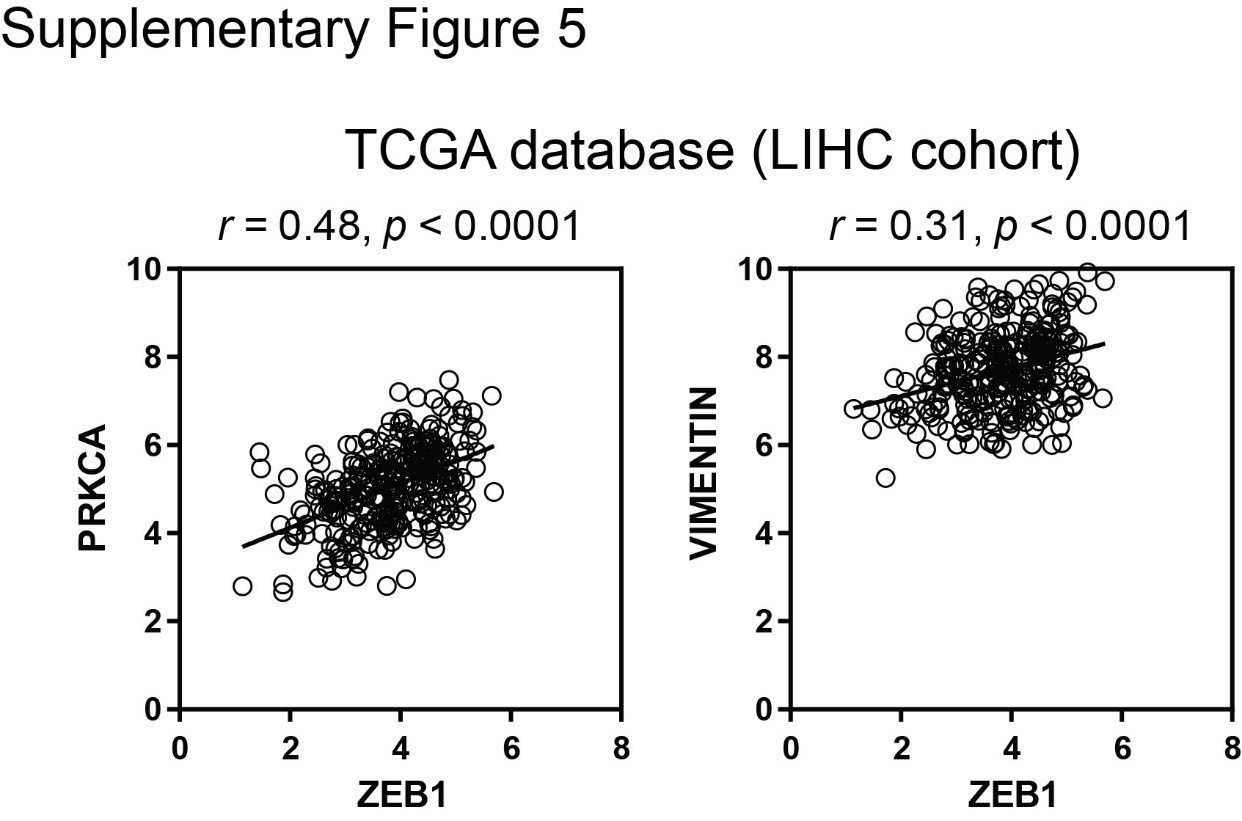


**Supplementary Figure 5:** *PRKCA* gene expression expression is associated with *ZEB1* in LIHC cohort (HCC) of Geneatlas database. Scatterplots of correlations between expression log-cpm for *ZEB1* and *PRKCA* genes. ZEB1-vimentin association was used to validate *ZEB1* gene expression marking M-HCC phenotype (right panel).


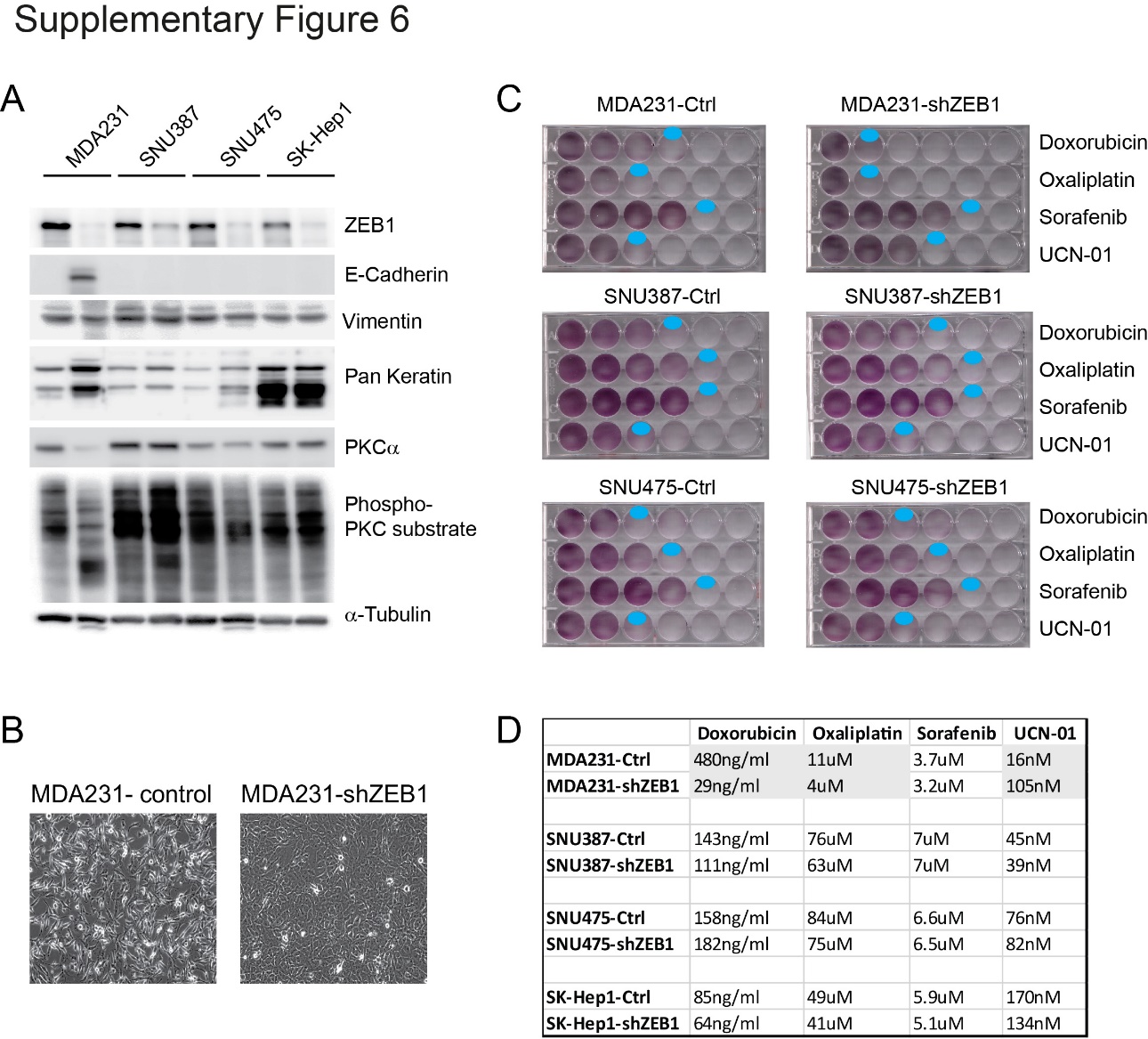


**Supplementary Figure 6:** Testing Mesenchymal-Epithelial Transition (MET) models of HCC with positive control (MDA231). **(A)** Three M-HCC (SNU387, SNU475 or SK-Hep1) cell lines and MDA231 cells, with or without shRNA targeting ZEB1 were tested for EMT markers (E-Cadherin, Vimentin, Pan-Keratin) as well as PKCα and PKC-substrate antibodies. Only MDA231-ZEB1 knock-down has undergone MET observed by inceased E-Cadherin and keratin expression. These cells also have reduced PKCα and PKC substrate phosphorylation. **(B)** MDA231 and ZEB1 kock-down MDA 231 cells are morphologically different. MDA231 represents a typical mesenchymal carcinoma cell line with spindle-like morphology where the ZEB1 knock-down version is epithelial, forming tight islands of cells. Drug resistance-sensitivity of all 8 cell lines were presented as crystal violet **(C)** or IC50 values **(D)**. Briefly, equal number of cells were seeded to 24 well plates and every column represents a concentration. The concentrations of drugs are: Doxorubicin: control, 16, 80, 400, 2000, 10.000ng/ml (5 fold increase increments), Oxaliplatin: control, 3,12.5, 50, 200, 800ug/ml (4 fold increase increments), Sorafenib: Control, 1.25, 2.5, 5, 10, 20uM (2 fold increase increments), UCN-01: Control, 6, 36, 200, 1200, 7200nM (6 fold increase increments). The point where a major decrease in viability was observed was marked with a blue spot. ZEB1 knock-down MDA231 cells became resistant to UCN-01 and sensitive to genotoxic agents. No change in Sorafenib sensitivity was observed in any cell. The IC50 curves obtained from Graphpad Prism software and presented as a chart **(D)**. Significant differences were indicated using grey shaded wells (only observed in MDA231) indicating M-HCC cells do not undergo MET upon ZEB1 knock-down.


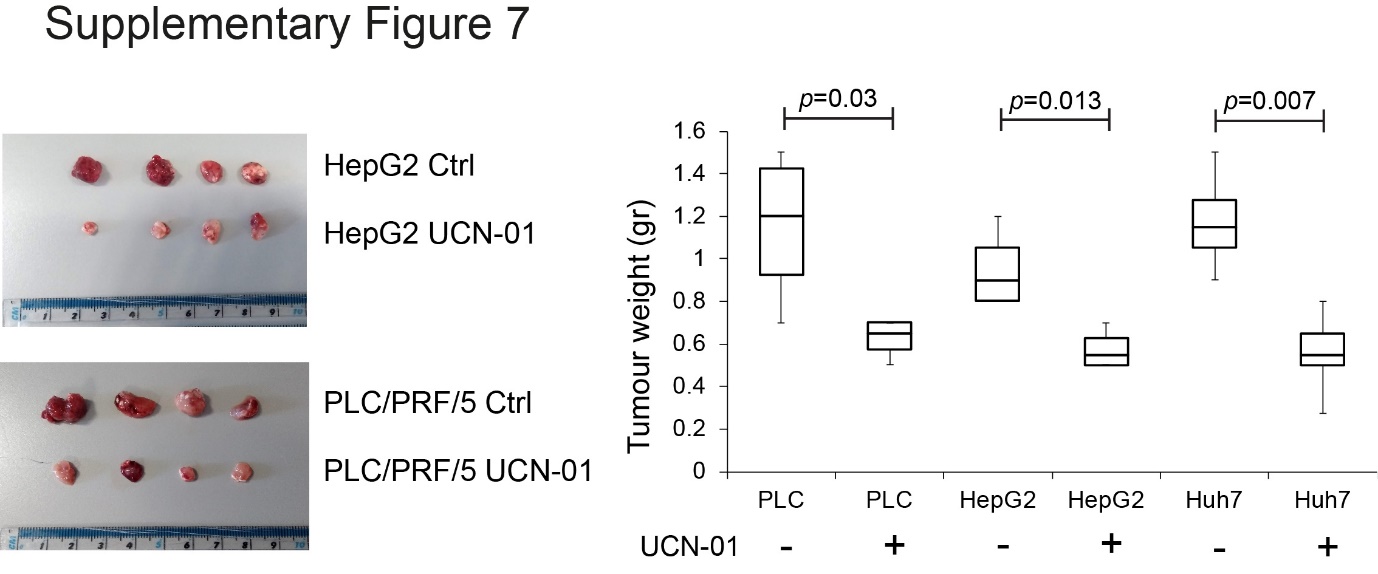


**Supplementary Figure 7:** Three E-HCC (Huh7, PLC/PRF/5 or HepG2) cell lines were injected (subcutaneously), allowed tumours to grow for 2 weeks and treated weekly with 2mg/kg UCN-01. Tumours were harvested 6 weeks after the intial treatment. Upon harvesting (left panels, Huh7 data was no shown) tumours were measured and weighed. UCN-01 induced significant regression of tumours (left panel).

**References**

1. Sayan AE, Griffiths TR, Pal R, Browne GJ, Ruddick A, Yagci T, et al. SIP1 protein protects cells from DNA damage-induced apoptosis and has independent prognostic value in bladder cancer. *Proc Natl Acad Sci U S A.* 2009;106(35):14884-9.

2. Oztas E, Avci ME, Ozcan A, Sayan AE, Tulchinsky E, and Yagci T. Novel monoclonal antibodies detect Smad-interacting protein 1 (SIP1) in the cytoplasm of human cells from multiple tumor tissue arrays. *Experimental and molecular pathology.* 2010;89(2):182-9.

3. Sayan AE, Stanford R, Vickery R, Grigorenko E, Diesch J, Kulbicki K, et al. Fra-1 controls motility of bladder cancer cells via transcriptional upregulation of the receptor tyrosine kinase AXL. *Oncogene.* 2012;31(12):1493-503.

4. Sayan BS, Sayan AE, Knight RA, Melino G, and Cohen GM. p53 is cleaved by caspases generating fragments localizing to mitochondria. *The Journal of biological chemistry.* 2006;281(19):13566-73.

5. Sayan AE, Sayan BS, Findikli N, and Ozturk M. Acquired expression of transcriptionally active p73 in hepatocellular carcinoma cells. *Oncogene.* 2001;20(37):5111-7.

6. Robinson MD, McCarthy DJ, and Smyth GK. edgeR: a Bioconductor package for differential expression analysis of digital gene expression data. *Bioinformatics.* 2010;26(1):139-40.

7. Ritchie ME, Phipson B, Wu D, Hu Y, Law CW, Shi W, et al. limma powers differential expression analyses for RNA-sequencing and microarray studies. *Nucleic Acids Res.* 2015;43(7):e47.
